# Bidirectional associations between parental feeding practices, infant appetitive traits and infant BMIz: a longitudinal cohort study

**DOI:** 10.1186/s12966-022-01392-z

**Published:** 2022-12-15

**Authors:** Alissa J Burnett, Elena Jansen, Jessica Appleton, Chris Rossiter, Cathrine Fowler, Elizabeth Denney-Wilson, Catherine G Russell

**Affiliations:** 1 Deakin University, Institute for Physical Activity and Nutrition (IPAN), School of Exercise and Nutrition Sciences, Geelong, Vic Australia; 2 Department of Psychiatry & Behavioral Sciences, Division of Child & Adolescent Psychiatry, Johns Hopkins University School of Medicine, Baltimore, USA; 3 Susan Wakil School of Nursing and Midwifery, University of Sydney, Sydney, NSW Australia; 4 Tresillian Family Care Centres, NSW, Australia; 5 School of Nursing and Midwifery, Faculty of Health, University of Technology Sydney, Sydney, NSW Australia; 6 Sydney Local Health District, Sydney, NSW Australia

**Keywords:** Appetitive traits, Infancy, Parent feeding, Weight, Cross-lagged, Child eating behaviours

## Abstract

**Background:**

Little is known about the pathways linking parent feeding practices with appetitive traits and BMIz throughout infancy. This study examined bidirectional associations between parental feeding practices, infant appetitive traits, and infant BMIz.

**Methods:**

Parents (*n* = 380) of infants aged less than 6 months at baseline reported their feeding practices (using the Feeding Practices and Structure Questionnaire (FPSQ) for infants and toddlers), infant appetitive traits (using the Baby Eating Behaviour Questionnaire) and infant BMIz (parent-reported) at three timepoints (< 6 months, ~ 9 months, ~ 12 months) up to 12 months of age. Cross-lagged models examined bidirectional associations between parent feeding practices, infant appetitive traits and infant BMIz.

**Results:**

There was strong continuity across the three timepoints for maternal feeding practices, infant appetitive traits, and infant BMIz. Infant food avoidance was prospectively associated with higher parental persuasive feeding. Infant BMIz was prospectively associated with higher parent-led feeding. Parent use of food to calm was prospectively associated with lower infant BMIz, and infant BMIz was prospectively associated with higher infant food approach. Feeding on demand was prospectively associated with lower infant food approach.

**Conclusion:**

This study highlights the complex associations between parental feeding practices, infant appetitive traits and infant BMIz. The study demonstrated that both child and parent effects are important, suggesting a need for tailored programs beginning in infancy to promote and support infant appetitive traits and parent feeding practices that support healthy development.

**Supplementary Information:**

The online version contains supplementary material available at 10.1186/s12966-022-01392-z.

## Background

Excess or rapid weight gain in infancy is associated with increased risk of overweight or obesity in early childhood [1], adolescence and adulthood [2]. While a number of risk factors has been identified (e.g. food approach appetitive traits) [3, 4], the processes (e.g. a change in parent behaviour in reaction to the child’s appetitive traits) that link these risk factors with excessive energy intake, and in turn overweight and obesity, are complex. We need to understand how identified risk factors casually explain overweight and obesity. There is recognition that a range of psychosocial and biological characteristics of parents and children and a number of different bidirectional and transactional processes are relevant to understanding the complex pathways to childhood overweight and obesity [5, 6]. Psychosocial processes can involve parent feeding practices and cognitions. Biological characteristics affecting such processes include infant/child temperament, neurocognitive functioning and genetics [5]. These psychosocial and biological processes, among others, have been linked to infant/child appetitive traits (defined as individual differences in patterns of behaviours and attitudes related to food and eating) [7–9] and weight, as well as to parent cognitions and feeding practices [5]. The current literature examining such processes has focussed largely on children older than two years [5]. Therefore, knowledge of the bidirectional and transactional processes affecting infant eating and weight will provide insights into the aetiology of overweight and obesity early in life, yet these processes may begin earlier, in infancy. The biopsychosocial model of the development of eating and weight in childhood proposes that an infant’s characteristics, appetitive traits and weight affect and are affected by parent feeding practices over time in complex pathways [5]. However, no studies have examined these processes in infancy to understand the origins of pathways linking early life risk factors to excess weight gain. Understanding these complex influences will inform programs aimed at preventing overweight and obesity early in infancy.

Presently, there is evidence from both cross-sectional and longitudinal studies on the relationships between parent feeding practices (defined as strategies that a parent engages in to influence their infant/child’s dietary intake [10]) and child/infant eating and weight outcomes. In these previous cross-sectional and longitudinal studies, there is evidence of relationships between parent feeding, child eating and child weight. Studies in children ranging from 2 to 9 years of age have shown that controlling and non-responsive feeding practices such as persuasive feeding are associated with higher child BMIz [11–13]. Parental feeding practices, such as pressure to eat and restriction, have also been shown to be associated with child appetitive traits (i.e., satiety responsiveness, food fussiness, emotional undereating, eating in the absence of hunger, food responsiveness and slowness in eating) [11, 14–18]. While, infant appetitive traits have also been shown to be associated with parental feeding practices [7] and weight outcomes [19]. However, the majority of these studies were conducted beyond the first year of life (2–9 years of age) [11–18], which does not allow for the examination of the early origins of these processes. Additionally, these studies were not designed to enable bidirectional and transactional effects to be examined and so the complex nature of these associations remains unclear. To establish the direction of association, it is necessary to use study designs with multiple waves of measurement of the same constructs, as child development theories suggest that parents adapt their parenting strategies to the child’s behaviour and weight [20]. Such studies will allow for the bidirectional and transactional associations to be examined.

Previous studies of children aged 1 to 9 years that have applied cross-lagged modelling (a technique used to examine two or more variables across different time points) to examine the bidirectional associations between feeding practices, child appetitive traits and/or BMIz have included children aged 1 to 9 years. These studies found both parent- [21–26] and child- [22, 24, 26–28] driven associations. With regard to the child-driven associations, studies found that parents use non-responsive feeding practices such as restriction, pressure to eat, and control in response to their child’s weight status [26–28]. Eating large amounts of food, slow eating, food refusal, and satiety responsiveness can evoke parent feeding practices such as structured meal timing, monitoring, pressure to eat, covert restriction, and overt restriction [22, 24]. With regard to the parent-driven associations, studies found that feeding practices such as pressure to eat, overt control and restriction of food predict higher child weight status at the next assessment point [22, 26]. Additionally, feeding practices such as using food as a reward for behaviour, covert restriction and family meal setting influence child appetitive traits including emotional overeating, food responsiveness and satiety responsiveness [21, 24, 25]. Finally, parental encouragement to eat has been shown to predict increased child enjoyment of food [25] and prompts to eat different food predicted eating in the absence of hunger [23]. However, all of these studies were conducted beyond the first year of life [21–28], which does not allow for examining these associations in early life when many processes originate.

Overall, while there is evidence that parents and children influence each other in relation to parent feeding, child eating and child weight, there are a number of limitations with the current evidence base. Previous cross-lagged studies (mentioned above) have only examined the associations between parental feeding practices and BMIz [26–28] or feeding practices and child appetitive traits [21, 22, 24, 25], while none has examine these simultaneously. There are no cross-lagged studies examining parent feeding, infant eating and infant weight during infancy to allow for the examination of early origins of these pathways [21–28]. However developmental literature stresses the importance of examining processes in infancy to understand their early origins [5]. Additionally, the time between waves of previous studies is between 1 and 4 years, in early years (0 to 2 years) it is important to assess infants and children more frequently as the stages of development are short. Examining these processes in infancy will enable us to further our understanding regarding the pathways to overweight and obesity that may begin during infancy [5]. It is important to explore the pathways that may link risk factors to child overweight and obesity to inform tailored early interventions including both the child and parents, rather than corrective interventions later in life.

Therefore, the aims of this exploratory study are to examine the directionality of associations between parental feeding practices (feeding on demand, food to calm, parent-led feeding and persuasive feeding), infant appetitive traits (food avoidance and food approach), and infant BMIz, and to examine the auto-regressive paths for each variable across timepoints. It is hypothesised, based on previous research in older children [21–28] and developmental literature [5], that persuasive and parent-led feeding practices will increase infant food approach appetitive traits, which will increase infant BMIz and the use of more restrictive feeding practices at later time points. Additionally, it is hypothesised that feeding practices, infant appetitive traits, and infant BMIz will increase at each time point.

## Methods

### Study design

This is a longitudinal cohort study of parents and their infant children. Participants completed three self-report surveys approximately three months apart, beginning when their infant was aged less than 6 months.

### Recruitment

Parents of infants aged less than 6 months were recruited between February 2016 and September 2016 via an early parenting support service in New South Wales, Australia: Tresillian Family Care Centres (https://www.tresillian.org.au/). Flyers and posters were displayed around the centres and nurses handed them to parents at appointments. If interested in the study, parents were provided with a Plain Language Statement (PLS) outlining details of the study and a hard copy of the survey or a link to an online survey. The completed survey was returned by parents to a sealed box at their Tresillian Centre and collected by research staff. Parents were also recruited via advertisements on the Tresillian Facebook group. If parents responded to the advertisement, they were linked to an electronic version of the PLS and survey (using the online platform SurveyGizmo). Participants were asked if they were willing to be contacted for a follow up survey; if they indicted yes, they were contacted via email 3 months after the first survey and again after a further 3 months. Eligible participants were parents of an infant less than 6 months of age, aged 18 years and over, and able to read and write in English. Participants were excluded from analysis if their infant was > 6 months of age at baseline, born at < 35 weeks gestation, < 2500 g birthweight, living outside Australia, or had a health condition that affected feeding. In appreciation of parents’ time, participants were able to enter a random draw for one of two iPads. No identifiable data were collected, thus removing any potential source of bias. Ethical approval was granted from the University of Technology Sydney Human Research Ethics Committee (REF NO. 2,015,000,528) and the Sydney Local Health District Human Research Ethics Committee (Protocol No X15-0233).

### Parent feeding practices

Parent feeding practices were measured using the Feeding Practices and Structure Questionnaire (FPSQ) for infants and toddlers [29]. This questionnaire can be used with parents who currently milk-feed their infant/child (18 items) or solid-feed their infant/child (34 items). At timepoints 1 and 2 the FPSQ milk feeding (FPSQ-M) version was administered. At timepoint 3 the parents were offered the FPSQ solid feeding (FPSQ-S) version if they were predominantly solid feeding (3 + meals or snacks per day). If they were feeding solids foods < 3 times per day, they were offered the FPSQ-M. These questionnaires measures four (FPSQ-M) or six (FPSQ-S) feeding practices: feeding on demand vs. feeding routine (e.g. “I let my baby decide when she/he would like to have a feed”), using food to calm (e.g., “I feed my baby to make sure that she/he does not get unsettled or cry”), persuasive feeding (e.g., “If my baby indicates she/he is not hungry, I try to get him to feed anyway”), parent-led feeding (e.g., “I feed my baby for a set time”), family meal environment, and using (non-) food rewards. The family meal environment and using (non-) food rewards are only included in the FPSQ-S and therefore not included in the current study. Each feeding practice consists of 4–7 items, which are rated on a five-point Likert scale ranging from 1 to 5 (never to always). The scores were averaged to obtain a single continuous score for each feeding practice. Both questionnaire versions were developed and validated in the current study sample and showed good internal reliability (Cronbach’s alphas: feeding on demand 0.87, food to calm 0.87, persuasive feeding 0.71, and parent-led feeding 0.79) [29].

### Infant appetitive traits

Infant appetitive traits were measured using the Baby Eating Behaviour Questionnaire (BEBQ), which originally asked parents to retrospectively rate their perceptions of their infant’s appetitive traits [30]. However, in the present study parents were asked about their infant’s current appetitive traits, therefore wording was changed to present tense (e.g., “my baby loved milk” was changed to “my baby loves milk”). The questionnaire contains questions on five appetitive traits: satiety responsiveness, slowness in eating, food responsiveness, enjoyment of food, and general appetite. Each appetitive trait consists of 3–6 items, with the exception of general appetite which only consists of one item. Responses were recorded on a five-point Likert scale ranging from 1 to 5 (never to always). The scores were averaged to obtain a continuous score for each appetitive trait. As conducted in previous research [19], the appetitive traits were combined into two categories: food approach (food responsiveness, enjoyment of food, general appetite) or food avoidance (satiety responsiveness and slowness in eating), resulting in a single continuous score for each category. The Cronbach’s alphas ranged between 0.75 and 0.77 at timepoint 1, 0.71 and 0.73 at timepoint 2, and 0.65 and 0.74 at timepoint 3 for slowness in eating, food responsiveness, and enjoyment of food, indicating moderate to good internal reliability for these appetitive traits. The Cronbach’s alphas were 0.62, 0.57, and 0.63 for satiety responsiveness at the three timepoints respectively.

### Infant body mass index z-score

At each timepoint, parents were asked to self-report their infant’s most recent weight, length and date of measurement based on their infant’s health record. Infant weight and length measurements are taken at regular health check-ups by a health professional (e.g., nurses, general practitioners) and recorded in their health record. To calculate the body mass index (BMI) z-scores, the World Health Organization’s age- and sex-specific growth charts were used [31]. BMI z-scores were used as a continuous variable.

### Statistical analysis

**Fig. 1:**
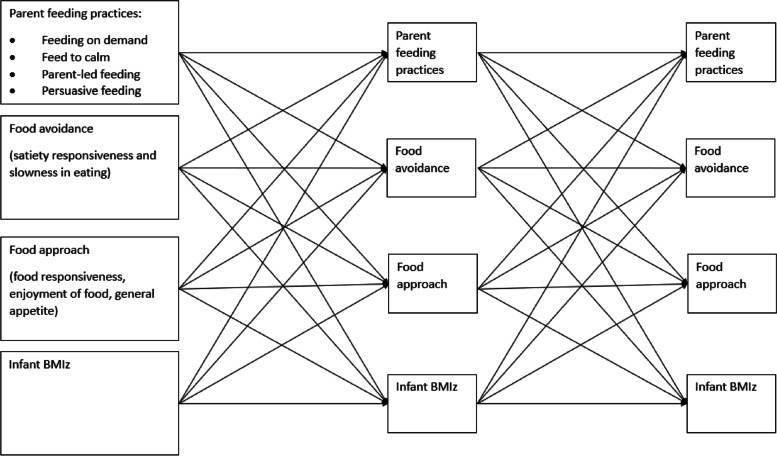
Cross-lagged model of parental feeding practices, infant appetitive traits, and infant BMIz

It was not possible to simultaneously include all variables in one model due to low statistical power. Therefore, as per Schreiber et al. [32] who recommend a ratio of ten participants to one parameter, four separate models (one model per feeding practice) were used to examine the longitudinal associations between parent feeding practices, infant appetitive traits and infant BMIz. Structural equation modelling was used to simultaneously estimate the cross-lagged associations between parental feeding practices (feeding on demand vs. feeding routine, using food to calm, persuasive feeding, and parent-led feeding), infant appetitive traits (food avoidance/food approach), and infant BMIz at each time point (Fig. 1). Auto-regressive paths for each variable were also examined across timepoints between parent feeding practices, infant appetitive traits, and infant BMIz at timepoint 1. This resulted in 32 pathways being estimated per model. Missing data were handled by estimating the parameters using the method of maximum likelihood which are iterative in nature [33]. All statistical analyses were conducted using Stata 16 [34]. The study was performed and reported following the strengthening the reporting of the observational studies in epidemiology (STROBE) guidelines [35].

## Results

In total, 445 parents provided some data; 65 participants were excluded, leaving 380 participants at T1, 178 at time 2, and 154 at time 3. The time between waves was a mean of 99 days (range 72–152) between T1 and T2, and 100 days (range = 54–182) between T2 and T3. Table 1 shows the descriptive characteristics of the participants. Just over half of the infants were male (54.2%), and the mean age was 3.22 months at T1, 6.51 months at T2 and 9.96 months at T3. Most parents were aged under 35 years of age (77.6%) and over half were university educated (57.6%).

**Table 1:** Sample characteristics

**Variable**	**All participants** **N (%) or mean (SD)**
Child age at T1 (months)	3.22 (1.56)
Child age at T2 (months)	6.51 (1.64)
Child age at T3 (months)	9.96 (1.64)
Parent sex	
Male	1 (0.3)
Female	372 (97.9)
Missing	7 (1.8)
Parent education	
University	219 (57.6)
No university	148 (39.0)
Missing	13 (3.4)
Parent age	
Under 29	149 (39.2)
30–34	146 (38.4)
Over 35	78 (20.5)
Missing	7 (1.8)
Country of birth	
Australia	318 (83.7)
Other	53 (14.0)
Missing	9 (2.8)
Child sex	
Male	206 (54.2)
Female	170 (44.7)
Missing	4 (1.1)

**Fig. 2:**
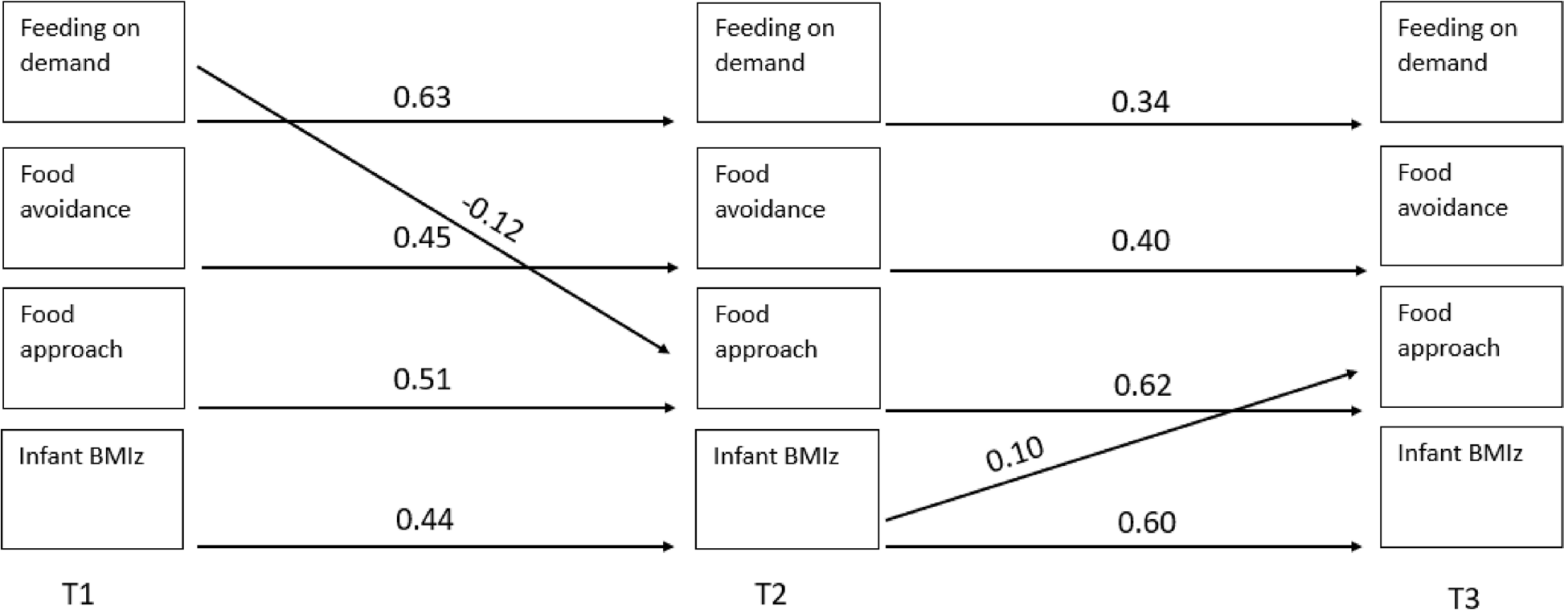
Statistically significant pathways in cross-lagged model examining longitudinal associations between parental feeding on demand, infant appetitive traits and infant BMIz

**Fig. 3:**
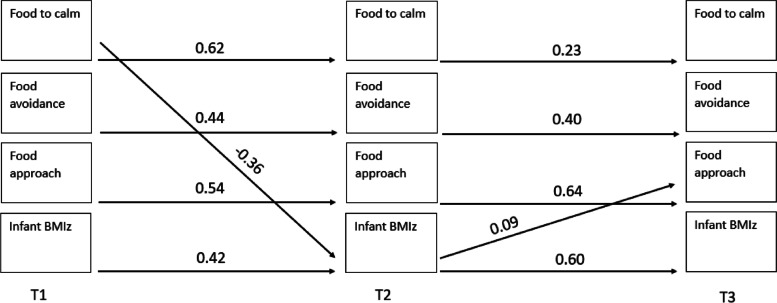
Statistically significant pathways in cross-lagged model examining longitudinal associations between parental food to calm, infant appetitive traits and infant BMIz

**Fig. 4:**
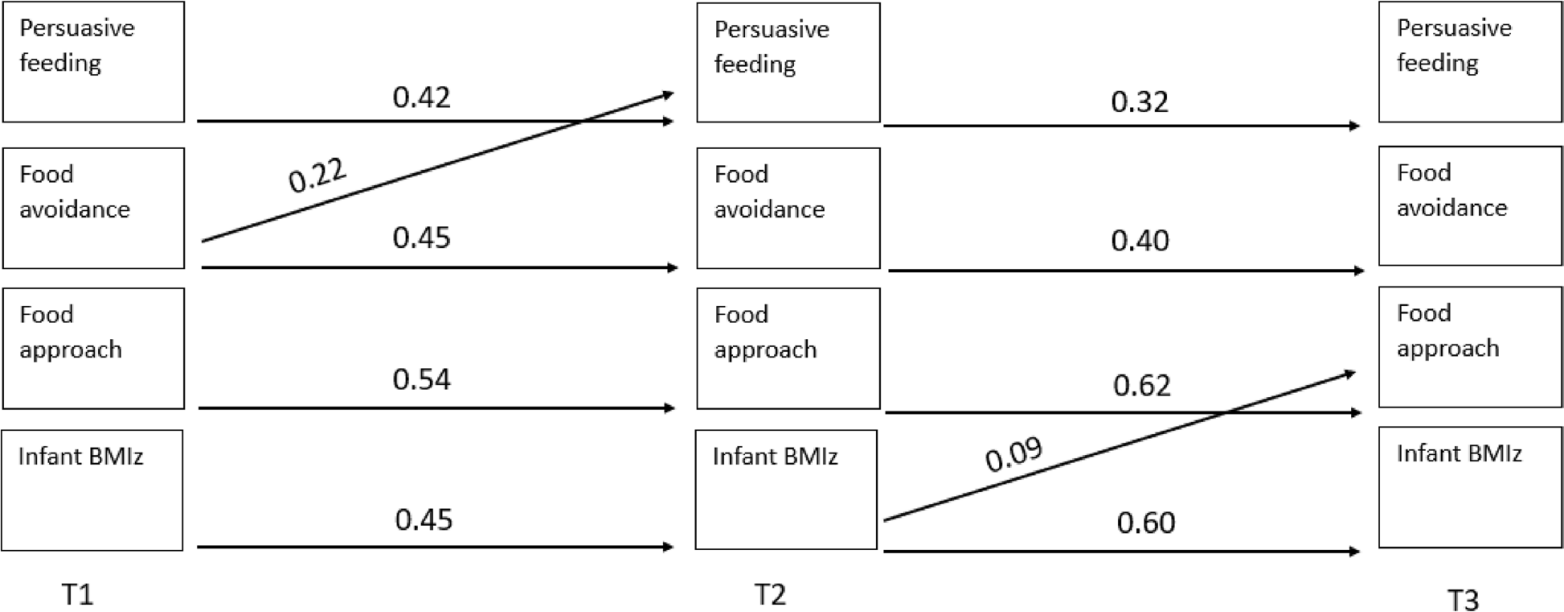
Statistically significant pathways in cross-lagged model examining longitudinal associations between parental persuasive feeding, infant appetitive traits and infant BMIz

**Fig. 5:**
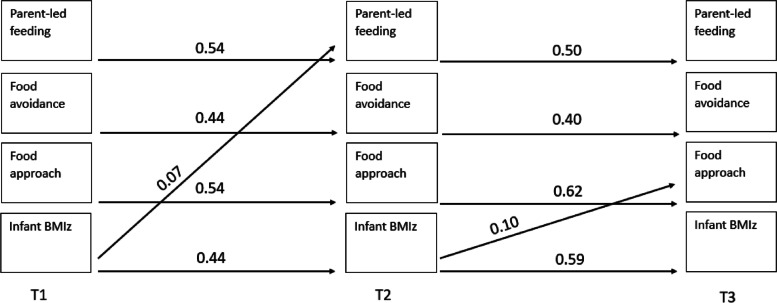
Statistically significant pathways in cross-lagged model examining longitudinal associations between parent-led feeding, infant appetitive traits and infant BMIz

Table 2 presents the means and standard deviations for parent feeding practices, infant appetitive traits, and infant BMIz at each timepoint. Of the 74 paths (14 auto-regressive and 60 cross-lagged) across four models, 19 (14 auto-regressive and 5 cross-lagged) were found to be significant. The results of all pathways tested are presented in Supplementary Tables 1–4. Figures 2, 3, 4 and 5 illustrates the significant associations between parent feeding practices, infant appetitive traits and infant BMIz at different time points.

**Table 2:** Means and standard deviations of the feeding practices, appetitive traits, and BMIz at timepoints 1, 2 and 3 (*n* = 380)

	Timepoint 1	Timepoint 2	Timepoint 3
Feeding on demand	2.02 (0.84)	2.22 (0.83)	3.49 (0.70)
Food to calm	2.54 (0.83)	2.41 (0.85)	1.83 (0.54)
Parent-led feeding	1.62 (0.69)	1.78 (0.67)	1.94 (0.68)
Persuasive feeding	1.94 (0.75)	1.84 (0.64)	2.44 (0.65)
Food avoidant appetitive traits^a^	2.57 (0.56)	2.30 (0.55)	2.16 (0.55)
Food approach appetitive traits^b^	3.44 (0.55)	3.34 (0.51)	3.36 (0.52)
BMIz	-0.28 (1.31)	0.28 (1.31)	0.47 (1.35)

^a^Food avoidant appetitive traits include satiety responsiveness and slowness in eating

^b^Food approach appetitive traits include food responsiveness, enjoyment of food, and general appetite

There were significant autoregressive paths (relationship between repeated measures over time) across timepoints for each maternal feeding practices, child appetitive traits, and BMIz with an increase from timepoint 1 to timepoint 2 (range 0.42–0.64) and from timepoint 2 to timepoint 3 (range 0.23–0.62), while food to calm and feeding on demand plateaued between the last two timepoints.

The cross-lagged paths suggest the associations were both child (appetitive traits or BMIz) driven (*n* = 3), and parent (parental feeding practices) driven (*n* = 2). Of the child-driven associations, two were associated with parent related outcomes, and one was associated with a child related outcome. That is, food avoidance at timepoint 1 was positively associated with persuasive feeding at timepoint 2. BMIz at timepoint 1 was positively associated with parent-led feeding at timepoint 2, and BMIz at timepoint 2 was positively associated with food approach at timepoint 3. Of the parent-driven associations, both were associated with child related outcomes. Feeding on demand at timepoint 1 was negatively associated with food approach at timepoint 2 and food to calm at timepoint 1 was negatively associated with BMIz at timepoint 2.

## Discussion

Findings from this novel longitudinal study of infant eating and parent feeding suggest that infants and parents influence each other in a range of complex ways. As expected, infant BMIz, infant appetitive traits and parent feeding practices were all significant components of the tested models. Firstly, the longitudinal findings show that in infancy, parents react to their infant’s appetitive traits and BMIz. Secondly, the findings suggest that parent feeding practices influence infant appetitive traits and BMIz. Thirdly, the findings showed that infant BMIz was associated with later infant appetitive traits. Finally, this study found there was continuity in maternal feeding practices, child appetitive traits, and infant BMIz across the three timepoints. However, there were also a number of non-significant relationships.

As hypothesised, the present study identified that infant food avoidant appetitive traits and higher infant BMIz predicted the use of non-responsive feeding practices at a later timepoint, such as persuasive feeding and parent-led feeding (i.e., the reliance, or lack thereof, on infant cues in regard to initiation and termination of feeding). Traditionally, the dominant perspective on infant eating and weight was a “parent-driven” perspective, with infants or children seen as passive recipients of parent feeding practices [36]. However, it is becoming recognised that parents and infants influence each other in contemporaneous, bidirectional and transactional ways [6, 21–28]. The findings of this study are supportive of this newer perspective, with parental feeding practices at times responding to their infant’s appetitive traits or BMIz, and these responses seen within the first 6 months of life. Previous research has also provided evidence for child effects on parent feeding practices: Costa et al. found that infant food refusal was associated with parental pressure to eat at a later timepoint [22], Jansen et al. found that satiety responsiveness was associated with structured meal timing, covert and overt restriction at a later timepoint [24], and several studies have shown that a higher child BMIz predicted controlling and restrictive feeding practices [26–28]. However, to our knowledge, this is the first study to show evidence of these associations in infancy. This study, together with previous research, shows that parents are sensitive to their infant or child’s BMIz across childhood and use particular feeding practices in response to this [28, 37]. However, previous research has found that these feeding practices may affect child weight and appetitive traits in unintended ways [38, 39], for instance diminishing the child’s responsiveness to internal satiety cues, which could then lead to the parent using further non-responsive feeding practices. Further longitudinal studies are needed to examine these associations over a longer period to understand how they evolve with development and affect child BMIz.

The present study also found that parental feeding practices can predict infant BMIz and appetitive traits. In line with our hypotheses, the present study found that parent feeding on demand (rather than by routine) was associated with lower infant food approach appetitive traits at a later timepoint, and that using food to calm at timepoint 1 predicted a lower BMIz at timepoint 2. This is inconsistent with previous literature in older children which shows that using food to calm or as a reward leads to unhealthy food intake and a higher weight status [40, 41]. At the age of the infants in wave 1 (average of 3 months), the food used to calm was likely either breastmilk or formula which may be associated with different patterns of weight development than using (non-core) foods to calm in older children. Food to calm may therefore be positively associated with weight status in children over one year of age and future research is needed to examine these associations in older infants and toddlers. Nevertheless, the findings are supportive of the idea that infants and parents influence each other in reciprocal ways, and this has relevance to appetitive traits and BMIz. Additionally, a transactional process was shown with food to calm at timepoint 1 being associated with lower BMIz at timepoint 2 which in turn was associated with higher infant food approach appetitive traits at timepoint 3. This is supportive of previous research in pre-school children which has found that if feeding is parent driven/led (such as covert restriction, encouragement to eat and using food as a reward) rather than child led, it predicts higher food approach appetitive traits in children (such as food responsiveness and enjoyment of food) [21, 24, 25]. The present study expands on previous work by demonstrating that these associations begin earlier than childhood; they can already be seen in infancy. To our knowledge, this is the first study to demonstrate a transactional process in infancy. However, while the findings were broadly supportive of the biopsychosocial transactional framework [6], it is important to note that this exploratory study tested a large number of pathways and not all pathways were significant. Therefore, further research is required, with a larger sample size, to confirm these findings.

As excepted, this study found the mean scores of feeding practices, some child appetitive traits and BMIz increase with age, which is consistent with previous studies [7, 9, 24, 42–44]. Two of the feeding practices (food to calm and feeding on demand) plateaued between the last two timepoints. However, this may be related to parents switching from milk feeding to solid feeding. Parents may be more inclined to follow a routine rather than feeding on demand or feeding to calm as infants get older and transition to solid foods. While only a small proportion of parents chose to fill out the solid feeding version, it is assumed that most children would be introduced to solids at this age (6 months) in line with typical Australian feeding practices [45]. Further research with a longer duration is needed to see if these feeding practices continue to plateau or increase again in the pre-school years. Food approach and BMIz had the strongest autoregressive pathways from timepoint 2 to timepoint 3, compared to the increase from timepoint 1 to timepoint 2.

These findings suggest that it could be important to develop tailored programs for parents during infancy directed at the pathways that lead to overweight and obesity (such as appetitive traits and parent feeding practices) rather than corrective interventions later in life. Such anticipatory programs have been undertaken in the past, these could be adopted and adapted with emerging insights into infancy [46]. However, further knowledge is needed before developing such programs. This study will aid in building the knowledge needed to develop intervention programs by establishing the relationships between appetitive traits, parent feeding practices and child BMIz status. Further longitudinal research should be conducted with a larger sample size to confirm these findings and allow the inclusion of more variables to explore additional pathways (such as parental cognitions and infant/child temperament) to further inform intervention programs [6].

This is the first study that we are aware of to incorporate appetitive traits, BMIz and feeding practices (appropriately measured for this age range) in one cross-lagged model in infancy, which has allowed for the novel examination of complex pathways to infant weight gain. However, as previously mentioned, there is a need for a larger cohort study tracking these behaviours from infancy into childhood and adolescence, to allow for the inclusion of additional variables, as per biopsychosocial models of eating and weight [6, 47–49]. Tracking these behaviours across the pre-school years, when we know food avoidance increases [50–52], and appetite self-regulation declines [53], will allow the examination of the possible multiple pathways and individual variations that predict BMIz status. Further, a larger sample size would also increase the generalisability of the findings and allow for the inclusion of time-invariant confounders. A limitation of this study includes the reliance on a single informant, which may lead to common method variance [54] and social desirability bias. Future studies would be strengthened with the use of multiple informants and multiple methods (including objective measures) to temper some of these measurement limitations. Additionally, the study also used the BEBQ to examine the child appetitive traits, which has limited data on validity and was originally developed for retrospective use with exclusively milk fed infants [30]; however, many studies have used the BEBQ in the same way as the current study [7, 55–57]. Lastly, there was a time difference between the date the parents completed the survey and the date of the last weight and length measurements by primary care providers (a mean of 18 days at T1, 24 days at T2 and 34 days at T3), and a different time length between each wave. Future cohort studies should measure all of the participants and all of the variables at the same time and reduce each timepoint to a smaller age range. It should also be noted that loss to follow-up over the 3 timepoints was high. However, the statistical approach used permits for and handles missing data, which reduces the risk of drop out bias.

## Conclusion

This study explored how infant appetitive traits, parent feeding practices and infant BMIz influence each other over time. Results suggested that effects appear to be both infant- and parent- driven, but also that there is continuity in appetitive traits, feeding practices, and infant BMIz over time. However, many of the expected pathways were not significantly associated, highlighting the need to better understand when and how infants and parents influence each other in feeding and eating.

Therefore, while these findings are broadly supportive of a transactional framework that suggests pathways in infant feeding, eating and BMIz emerge early in life, further research with more sophisticated measures and that follow infants into toddlerhood and beyond will provide greater insights into the pathways explaining the development of eating behaviours and BMIz that can form the basis of early, targeted intervention programs.

## Supplementary Information

**Additional file 1: Supplementary Table 1.** The associations between feeding on demand, appetitive traits and infant BMI (*n*=380). **Supplementary Table 2.** The associations between food to calm, appetitive traits and infant BMI (*n*=380). **Supplementary Table 3.** The associations between persuasive feeding, appetitive traits and infant BMI (*n*=380). **Supplementary Table 4**. The associations between parent-led feeding, appetitive traits and infant BMI (*n*=380).

## Data Availability

The datasets used and/or analysed during the current study are available from the corresponding author on reasonable request.

## Abbreviations

BMI: Body mass index
BMIz: Body mass index z-score
BEBQ: Baby Eating Behaviour Questionnaire
