# Supplementary material for: Bidirectional associations between parental feeding practices, infant appetitive traits and infant BMIz: a longitudinal cohort study
Source: Int J Behav Nutr Phys Act. 2022 Dec 15;19:153. doi: 10.1186/s12966-022-01392-z (PMC9753278; doi:10.1186/s12966-022-01392-z)
Supplement: Supplementary file 1 — Additional file 1: Supplementary Table 1. The associations between feeding on demand, appetitive traits and infant BMI (n=380). Supplementary Table 2. The associations between food to calm, appetitive traits and infant BMI (n=380). Supplementary Table 3. The associations between persuasive feeding, appetitive traits and infant BMI (n=380). Supplementary Table 4. The associations between parent-led feeding, appetitive traits and infant BMI (n=380). [file 12966_2022_1392_MOESM1_ESM.doc]

**Supplementary Table 1: The associations between feeding on demand, appetitive traits and infant BMI (n=380)**

|  | FAv1 | FAp1 | BMI1 | FD2 | FAv2 | FAp2 | BMI2 | FD3 | FAv3 | FAp3 | BMI3 |
| --- | --- | --- | --- | --- | --- | --- | --- | --- | --- | --- | --- |
| FD1 | 0.02 | -0.10*** | 0.14* | 0.63*** | -0.01 | -0.12** | 0.06 |  |  |  |  |
| FAv1 |  | -0.08*** | -0.06 | 0.00 | 0.45*** | -0.02 | -0.24 |  |  |  |  |
| FAp1 |  |  | 0.01 | 0.15 | -0.10 | 0.51*** | 0.15 |  |  |  |  |
| BMI1 |  |  |  | 0.03 | 0.02 | 0.01 | 0.44*** |  |  |  |  |
| FD2 |  |  |  |  |  |  |  | 0.34*** | 0.04 | -0.02 | 0.16 |
| FAv2 |  |  |  |  |  |  |  | 0.05 | 0.40*** | -0.04 | -0.16 |
| FAp2 |  |  |  |  |  |  |  | 0.10 | 0.00 | 0.62*** | -0.15 |
| BMI2 |  |  |  |  |  |  |  | 0.05 | -0.04 | 0.10*** | 0.60*** |

*p value = <0.05 **p value = <0.01 ***p value = <0.001

Abbreviations: FD = Feeding on demand, FAv = Food avoidance, FAp = Food approach, BMI = Body mass index

**Supplementary Table 2: The associations between food to calm, appetitive traits and infant BMI (n=380)**

|  | FAv1 | FAp1 | BMI1 | FC2 | FAv2 | FAp2 | BMI2 | FC3 | FAv3 | FAp3 | BMI3 |
| --- | --- | --- | --- | --- | --- | --- | --- | --- | --- | --- | --- |
| FC1 | 0.02 | 0.11*** | 0.01 | 0.62*** | 0.05 | 0.01 | -0.36** |  |  |  |  |
| FAv1 |  | -0.08*** | -0.07 | 0.07 | 0.44*** | -0.02 | -0.19 |  |  |  |  |
| FAp1 |  |  | 0.01 | 0.11 | -0.11 | 0.54*** | 0.21 |  |  |  |  |
| BMI1 |  |  |  | 0.03 | 0.03 | -0.01 | 0.42*** |  |  |  |  |
| FC2 |  |  |  |  |  |  |  | 0.23*** | 0.00 | -0.04 | -0.04 |
| FAv2 |  |  |  |  |  |  |  | 0.04 | 0.40*** | -0.03 | -0.16 |
| FAp2 |  |  |  |  |  |  |  | 0.06 | -0.02 | 0.64*** | -0.20 |
| BMI2 |  |  |  |  |  |  |  | 0.01 | -0.03 | 0.09** | 0.60*** |

*p value = <0.05 **p value = <0.01 ***p value = <0.001

Abbreviations: FC = Food to calm, FAv = Food avoidance, FAp = Food approach, BMI = Body mass index

**Supplementary Table 3: The associations between persuasive feeding, appetitive traits and infant BMI (n=380)**

|  | FAv1 | FAp1 | BMI1 | PF2 | FAv2 | FAp2 | BMI2 | PF3 | FAv3 | FAp3 | BMI3 |
| --- | --- | --- | --- | --- | --- | --- | --- | --- | --- | --- | --- |
| PF1 | 0.08*** | 0.00 | -0.05 | 0.42*** | -0.04 | -0.04 | -0.09 |  |  |  |  |
| FAv1 |  | -0.08*** | -0.07 | 0.22** | 0.45*** | -0.01 | -0.21 |  |  |  |  |
| FAp1 |  |  | 0.01 | 0.05 | -0.10 | 0.54*** | 0.10 |  |  |  |  |
| BMI1 |  |  |  | 0.03 | 0.02 | -0.01 | 0.45*** |  |  |  |  |
| PF2 |  |  |  |  |  |  |  | 0.32** | -0.01 | 0.02 | 0.36 |
| FAv2 |  |  |  |  |  |  |  | 0.18 | 0.40*** | -0.04 | -0.34 |
| FAp2 |  |  |  |  |  |  |  | 0.04 | -0.02 | 0.62*** | -0.22 |
| BMI2 |  |  |  |  |  |  |  | 0.05 | -0.04 | 0.09** | 0.60*** |

*p value = <0.05 **p value = <0.01 ***p value = <0.001

Abbreviations: PF = Persuasive feeding, FAv = Food avoidance, FAp = Food approach, BMI = Body mass index

**Supplementary Table 4: The associations between parent-led feeding, appetitive traits and infant BMI (n=380)**

|  | FAv1 | FAp1 | BMI1 | PLF2 | FAv2 | FAp2 | BMI2 | PLF3 | FAv3 | FAp3 | BMI3 |
| --- | --- | --- | --- | --- | --- | --- | --- | --- | --- | --- | --- |
| PLF1 | 0.07** | -0.05* | -0.01 | 0.54*** | 0.01 | -0.07 | 0.20 |  |  |  |  |
| FAv1 |  | -0.08*** | -0.07 | 0.05 | 0.44*** | -0.01 | -0.26 |  |  |  |  |
| FAp1 |  |  | 0.01 | 0.13 | -0.10 | 0.54*** | 0.15 |  |  |  |  |
| BMI1 |  |  |  | 0.07* | 0.02 | -0.01 | 0.44*** |  |  |  |  |
| PLF2 |  |  |  |  |  |  |  | 0.50*** | 0.00 | 0.02 | 0.33 |
| FAv2 |  |  |  |  |  |  |  | 0.17 | 0.40*** | -0.04 | -0.19 |
| FAp2 |  |  |  |  |  |  |  | 0.11 | -0.03 | 0.62*** | -0.18 |
| BMI2 |  |  |  |  |  |  |  | 0.00 | -0.04 | 0.10** | 0.59*** |

*p value = <0.05 **p value = <0.01 ***p value = <0.001

Abbreviations: PLF = Parent-led feeding, FAv = Food avoidance, FAp = Food approach, BMI = Body mass index
